# Climate Change and Cardiovascular Health: Environmental Stressors, Mechanistic Insights, and Clinical Perspectives

**DOI:** 10.31083/RCM40069

**Published:** 2025-10-23

**Authors:** Tingting Lv, Qing Liu, Yifei Wang, Ping Zhang

**Affiliations:** ^1^Department of Cardiology, Beijing Tsinghua Changgung Hospital, School of Clinical Medicine, Tsinghua University, 102218 Beijing, China; ^2^Clinical Medical College, Qinghai University, 810000 Xining, Qinghai, China; ^3^Department of General Practice, Suining Central Hospital, 629000 Suining, Sichuan, China

**Keywords:** climate change, cardiovascular health, air pollution, heatwaves, environmental stressors

## Abstract

Climate change poses a significant threat to cardiovascular health through the combined effects of extreme temperatures, air pollution, and extreme weather events. Short-term heat exposure raises mortality risk by 3.80%, while long-term exposure to particulate matter (PM_2.5_, with an aerodynamic diameter of ≤2.5 micrometers) increases cardiovascular mortality by 11–20%. Key mechanisms include thermoregulatory stress, inflammation, autonomic nervous system dysfunction, prothrombotic state, and psychosocial stress. Vulnerable groups, such as older individuals and those with cardiovascular diseases, also face a higher risk. Epidemiological studies have shown that for every one-standard-deviation increase in the number of days with excess heat factor, the overall mortality risk rises by 3.80%. Proposed interventions include high-efficiency particulate air (HEPA) purifiers, optimized cooling centers, and low-emission zones. However, key research gaps remain in the effects of multi-stressors, protection strategies, exposure assessment, and climate-driven disease projections. Multidisciplinary collaboration is crucial for mitigating climate-related cardiovascular risks. This review provides a comprehensive overview of the current situation regarding climate change and cardiovascular health, summarizing the results of epidemiological, pathological mechanisms, and policy research.

## 1. Introduction

Climate change has become one of the important factors threatening health. Driven by increased greenhouse gas emissions and intensified human activities, the Earth’s climate is undergoing unprecedented changes, characterized by more frequent and severe extreme weather events such as global warming, heat waves, heavy rainfall, and hurricanes [1, 2]. Meanwhile, cardiovascular diseases (CVDs) remain the leading cause of death worldwide [3, 4]. Emerging evidence indicates that climate-related environmental stressors—including extreme temperatures, air pollution, wildfire smoke, and psychological stress linked to climate disasters—adversely affect both acute and chronic cardiovascular health [5, 6, 7]. These findings suggest a complex interplay between climate change and cardiovascular morbidity and mortality, mediated by diverse biological mechanisms that are yet to be fully understood.

**Fig. 1.**
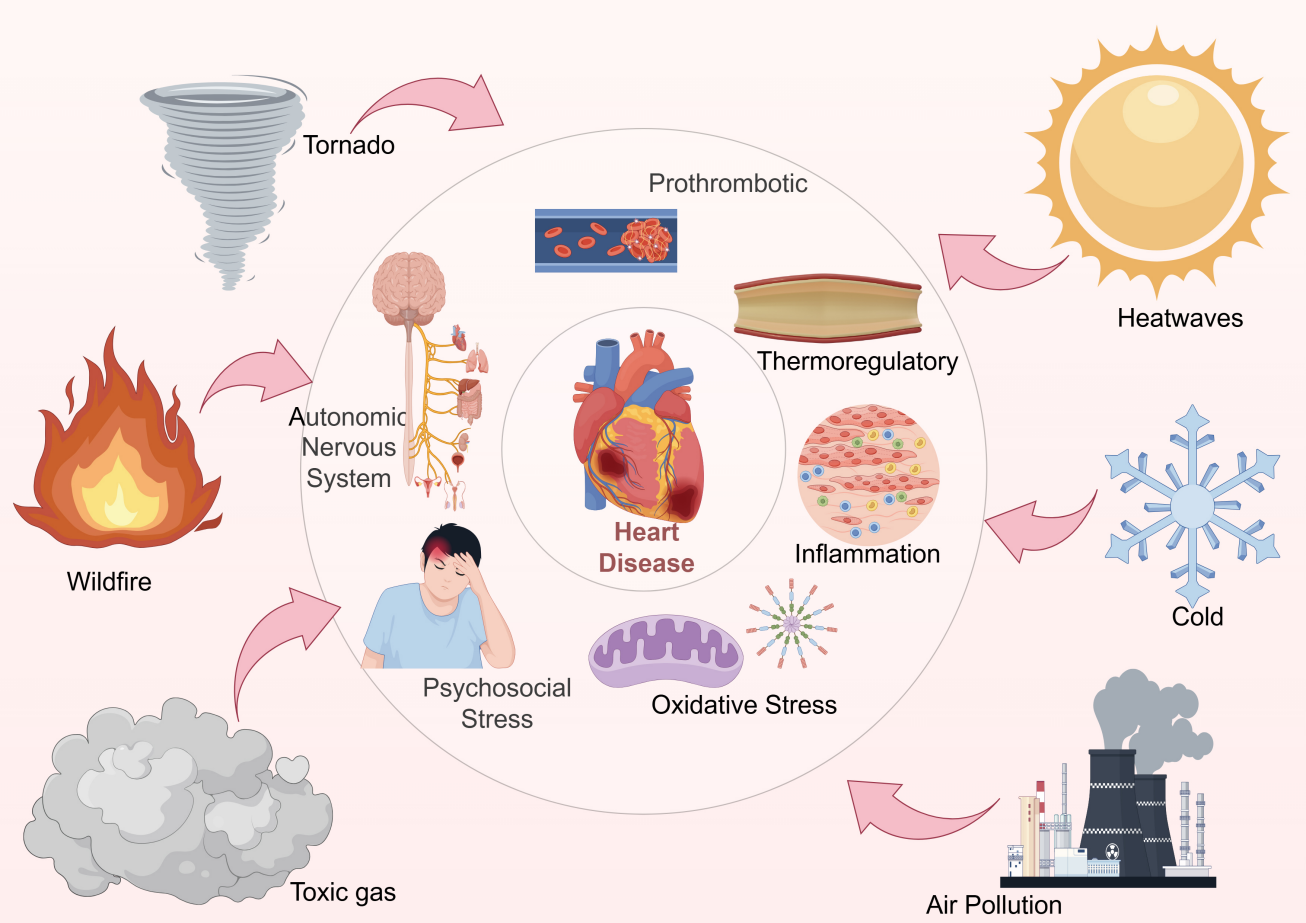
**Schematic overview of the relationship between climate change and cardiovascular outcomes**. This figure illustrates the pathways linking various climate change factors (e.g., extreme temperatures, air pollution) to CVD risks. This figure was created using Figdraw.

This review aims to systematically examine the environmental stressors associated with climate change and elucidate the underlying pathophysiological mechanisms contributing to cardiovascular events. Additionally, we summarize key epidemiological findings and evaluate current public health strategies intended to mitigate cardiovascular risks in the context of a changing climate. By integrating evidence from multiple disciplines, this article seeks to provide novel insights to inform targeted interventions and policy development. Fig. 1 illustrates the conceptual framework linking climate change, environmental stressors, biological pathways, and cardiovascular outcomes.

## 2. Climate-Related Environmental Stressors

Previous studies have found that 9.4% of deaths each year are related to non-optimal temperatures, with 8.5% related to low temperatures and 0.9% related to high temperatures [8]. In addition, air pollution, extreme high temperatures, and severe weather patterns will increase the incidence rate and hospitalization rate of CVDs [9].

### 2.1 Heatwaves

Heatwaves are associated with increased cardiovascular mortality and incidence rates. High ambient temperatures trigger thermoregulatory responses such as peripheral vasodilation and increased sweating, which in turn lead to dehydration, reduced plasma volume, and elevated heart rate. These changes increase cardiac workload and may precipitate acute events in vulnerable individuals. Exposure to extremely high or low temperatures increased the risk of ischemic heart disease, heart failure, arrhythmia, and myocardial infarction (MI) [10, 11]. For example, a Pan-Latin American analysis across 326 cities found that each 1 °C increase above the 95th-percentile temperature was associated with a 5.7% increase in all-cause mortality, while each 1 °C decrease below the 5th percentile led to a 3.4% rise [12]. In addition, previous studies have shown that high temperatures can cause dehydration, increased heart rate, and changes in blood pressure regulation, ultimately leading to acute cardiovascular events such as myocardial infarction and stroke [13].

### 2.2 Cold Exposure

Cold exposure exerts distinct pathophysiological effects on the cardiovascular system. Low temperatures lead to peripheral vasoconstriction, sympathetic nervous system activation, and elevated blood pressure, which together increase myocardial oxygen demand and blood viscosity. These changes heighten the risk of thrombotic events such as MI and stroke. Research has found that there is a J-shaped relationship between temperature and the mortality rate of CVDs. The optimal survival temperature is approximately 21–22 °C (near the median), and the risk of death is the lowest within this range. Recent studies have indicated that extremely low temperatures are associated with cardiovascular mortality in people with cerebrovascular diseases, males, married people, and people above the age of 65 years. In addition, the impact of cold temperature on CVD was delayed by 2–6 days and persisted for 4–10 days [14]. These results highlight the need for targeted protection during winter weather events.

### 2.3 Air Pollution

Air pollutants—including particulate matter (PM_2.5_), nitrogen dioxide (NO_2_), and ozone (O_3_)—are well-documented contributors to cardiovascular pathophysiology. Epidemiological studies have shown that environmental air pollution, especially inhalable fine particulate matter, is closely related to cardiovascular incidence rate and mortality [15]. Besides, PM_2.5_ could cause a series of adverse health effects such as cancer and lung diseases, metabolic disorders, and adverse birth outcomes. Short-term exposure, from several hours to days, increases the risk of myocardial infarction, stroke, heart failure, arrhythmia, and sudden death, and the risk of cardiovascular events is higher with the increase of exposure time [16]. A meta-analysis found that for every 10 µg/m^3^ increase in short-term exposure to PM_2.5_, the risk of all-cause cardiovascular mortality increased by 0.64%–1.00%, while for long-term exposure, the risk increased by as much as 11% to 20%. For every 10 µg/m^3^ increase in short-term exposure to NO_2_, the all-cause rate is 0.88%–1.62%, and for long-term exposure, it reaches 3%–23% [17].

### 2.4 Extreme Weather Events

Extreme weather events refer to rare or severe meteorological phenomena, including hurricanes, floods, heatwaves, cold spells, and droughts. Emerging evidence indicates that hurricanes elevate cardiovascular risk profiles through mechanisms including compromised healthcare infrastructure and disrupted service delivery. The Preparedness to Reduce Exposures and Diseases Post-hurricanes and Augment Resilience (PREPARE) cohort study in Puerto Rico followed up 364 obese adults (age 40–65 years) pre- and post-hurricanes Irma and Maria, revealing a significant increase in hypertension prevalence from 39.3% to 44.8% (odds ratio (OR) = 2.2, 95% Confidence interval (CI) 1.2–3.9) [18]. A 17-year (1999–2016) study in Florida, USA, which included over 3.5 million Medicare hospitalizations found that the risk of cardiovascular hospitalizations decreased on the day of tropical cyclone landfall (relative risk (RR) = 0.74; 95% CI 0.70–0.79), but rebounded and increased on the 3rd to 6th day, especially in high poverty communities, where the cumulative risk of cardiovascular hospitalizations increased to 1.45 on the 10th day (95% CI 1.14–1.85) [19]. In addition, A retrospective cohort study encompassing 11,801,527 Medicare beneficiaries aged ≥65 years revealed that flood exposure was associated with 4.8% (Internal Rate of Return (IRR) = 1.05; 95% CI 1.04–1.05) and 7.4% (IRR = 1.07; 95% CI 1.07–1.08) increases in emergency department visits and unplanned hospitalizations, respectively, relative to pre-exposure periods [20]. On the other hand, a nationwide case-crossover study of 120,380 hospitalized MI patients during cold seasons (October to March) in Sweden demonstrated that cold waves (≥2 consecutive days with location-specific daily mean temperatures below the 10th percentile) were associated with elevated MI risks during lag periods of 2–6 days. The adjusted odds ratios for total MI, non-ST-segment elevation MI (NSTEMI), and ST-segment elevation MI (STEMI) onset were 1.077 (95% CI 1.037–1.120), 1.069 (95% CI 1.020–1.119), and 1.095 (95% CI 1.023–1.172), respectively [21]. In addition, under the dual conditions of high temperature and drought, the risk of ischemic stroke increases (RR = 1.20, 95% CI: 1.03–1.40) [22]. However, contrary to the impact of hurricanes, tornadoes do not seem to increase the prevalence of cardiovascular events [23]. The above research confirms that extreme weather significantly affects cardiovascular health at the spatiotemporal scale through multiple mechanisms, highlighting the importance of post-disaster public health interventions, medical service resilience, and climate adaptation strategies.

### 2.5 Wildfire Smoke

Wildfires generate large amounts of smoke that contains a mixture of fine particulate matter and toxic gases [24, 25]. Research has identified an interaction between the duration of smoke events and environmental temperatures, suggesting that wildfire exposure increases the risk of cardiovascular events, including ischemic heart disease and heart failure [26]. In addition to particulate matter, toxic gases such as carbon monoxide (CO) and NO_2_ also contribute significantly to cardiovascular risk. CO impairs oxygen delivery by forming carboxyhemoglobin, thereby exacerbating myocardial ischemia. NO_2_ can induce endothelial dysfunction, oxidative stress, and heightened systemic inflammation. Notably, these gases may act synergistically with PM_2.5_ to potentiate adverse cardiovascular effects, especially in susceptible populations [27, 28]. Table 1 (Ref. [10, 11, 12, 13, 14, 15, 16, 17, 18, 19, 20, 21, 22, 23, 24, 25, 26, 27, 28]) summarizes the impact of some climate-related environmental stressors on cardiovascular health.

**Table 1.** **Summary of climate-related environmental stressors and cardiovascular health effects**.

Stressor type	Exposure characteristics	Cardiovascular effects	Key mechanisms	Key references
Heatwaves	Sudden temp >35 °C, multiple days	MI, HF, arrhythmia, mortality	Dehydration, thermoregulation overload	Alahmad B *et al*. 2023 [10]; Xu R *et al*. 2023 [11]; Kephart JL *et al*. 2022 [12]; Hong L *et al*. 2023 [13]
Cold exposure	Temp <10th percentile	MI, stroke, BP surge	Vasoconstriction, BP, blood viscosity	Zhang W *et al*. 2021 [14]
Air pollution	Short- and long-term	MI, stroke, atherosclerosis	Inflammation, ROS, and autonomic dysregulation	Davidovich L and Saldiva P 2019 [15]; Rajagopalan S *et al*. 2020 [16]; de Bont J *et al*. 2022 [17]
Extreme weather	Hurricanes, floods, cold waves	ED visits, HTN, MI, mortality	Access disruption, physical/mental trauma	Martínez-Lozano M *et al*. 2023 [18]; Burrows K *et al*. 2023 [19]; Wettstein ZS *et al*. 2025 [20]; Ni W *et al*. 2024 [21]; Zhang H *et al*. 2024 [22]; Silva-Palacios F *et al*. 2015 [23]
Wildfire smoke	PM_2.5_+CO/NO_2_+heat synergy	HF, IHD, mental health burden	Oxidative stress, endothelial dysfunction	Aguilera R *et al*. 2021 [24]; Stowell JD *et al*. 2019 [25]; Heaney A *et al*. 2022 [26]; Warnakulasuriya T *et al*. 2024 [27]; Zhang D *et al*. 2023 [28]

MI, myocardial infarction; HF, heart failure; BP, blood pressure; HTN, Hypertension; IHD, Ischemic Heart Disease; ROS, Reactive Oxygen Species; PM_2.5_, particulate matter; CO, carbon monoxide; NO_2_, nitrogen dioxide.

## 3. Mechanistic Pathways Linking Environmental Exposure to Cardiovascular Risk

### 3.1 Thermoregulatory and Hemodynamic Stress

When ambient temperature surpasses human thermoregulatory capacity, the body initiates systemic hemodynamic redistribution and compensatory cardiovascular responses to facilitate thermolytic processes, at the expense of imposing considerable hemodynamic strain. To facilitate thermoregulatory blood volume redistribution towards cutaneous surfaces for heat dissipation, cutaneous microvascular perfusion may augment to 4–8 L/min, thereby inducing systemic reductions in peripheral vascular resistance that pose hemodynamic challenges to arterial pressure homeostasis [29, 30]. A 0.9 °C elevation in core body temperature induces a median heart rate escalation of 27 beats per minute (bpm), with concurrent elevations in cardiac output and systemic oxygen consumption alongside attenuated systolic blood pressure decline, collectively demonstrating augmented myocardial workload during thermal stress exposure. In a controlled heat exposure study at 40 °C and 9% relative humidity, 20 young adults (19–31 years) and 39 older adults (61–78 years) underwent 9 h of resting exposure. The elderly group exhibited a 0.3 °C higher core temperature at 6 h compared to the younger group. Moreover, they experienced greater reductions in mean arterial pressure (MAP) and slower heart rate recovery, indicating diminished cardiovascular compensatory responses. Quantitative comparisons revealed that older adults had approximately 30% lower sweat rates and significantly blunted cutaneous vasodilation, reflecting impaired thermoregulatory capacity through both evaporative and convective heat loss mechanisms [31]. These limitations are particularly critical under extreme heat conditions. Excessive sweating leads to a 5–10% decrease in plasma volume, a reduction in central venous pressure, and a corresponding decline in stroke volume. To maintain cardiac output, the body compensates by increasing heart rate. This cascade—beginning with dehydration, followed by reduced preload and reflex tachycardia—not only increases myocardial oxygen demand but may also precipitate hypotensive syncope, especially in heat-sensitive individuals.

### 3.2 Systemic Inflammation and Oxidative Stress

Environmental exposure can induce systemic inflammatory response and oxidative stress, damage the cardiovascular system, and promote disease occurrence. Fine PM_2.5_ can activate NF-κB and other inflammatory signaling pathways in alveolar macrophages and vascular endothelial cells, releasing various pro-inflammatory cytokines and chemokines, thereby triggering systemic low-grade chronic inflammation [32, 33, 34, 35]. Among the upregulated inflammatory mediators, interleukin-6 (IL-6) and tumor necrosis factor-*alpha* (TNF-α) play different roles. IL-6 is closely associated with chronic low-grade inflammation and serves as a key driver of hepatic C-reactive protein (CRP) production, thereby acting as a bridge between air pollution exposure and subclinical atherosclerosis. In contrast, TNF-α exerts potent pro-inflammatory and cytotoxic effects, contributing to endothelial dysfunction, myocardial remodeling, and cardiomyocyte apoptosis. While IL-6 predominantly mediates systemic inflammatory responses, TNF-α is more strongly implicated in acute vascular injury and plaque instability. These differences suggest that air pollution–induced cardiovascular risk may be the result of synergistic inflammatory pathways involving both systemic and vascular compartments. A prospective cohort study from Chicago (n = 641) found that for every 1 µg/m^3^ increase in cumulative PM_2.5_ exposure (1–36 months average), linear increases in monocyte chemoattractant protein-1 (MCP-1), TNF-α, and IL-6 levels were observed [36]. These inflammatory mediators can promote macrophage migration into atherosclerotic plaque, endothelial cell activation, and vascular smooth muscle cell proliferation, thereby accelerating the process of atherosclerosis and increasing the risk of acute cardiovascular events. Metal ions, organic carbon, and transition metal components in PM_2.5_ can generate excessive reactive oxygen species (ROS) at the mitochondrial and cell membrane levels, depleting the activity of intracellular antioxidant enzymes such as superoxide dismutase and glutathione peroxidase, leading to lipid peroxidation and protein and DNA damage. A longitudinal cohort study in Chiang Mai, Thailand (n = 25 healthy adults) comparing urinary biomarkers between high-pollution (PM_2.5_ ∼ 67 µg/m^3^) and low-pollution (PM_2.5_ ∼ 7 µg/m^3^) seasons demonstrated significant elevation of malondialdehyde (MDA), 8-isoprostaglandinF2α (8-epi-PGF2α), and 1-hydroxypyrene (1-OHP) levels during high-exposure periods, confirming intensified systemic oxidative stress [37]. On the other hand, ROS accumulation induces vascular endothelial dysfunction, facilitates low-density lipoprotein oxidation and thrombogenesis, while concurrently amplifying inflammatory responses through NOD-like receptor family pyrin domain containing 3 (NLRP3) inflammasome activation pathways.

### 3.3 Autonomic Nervous System Dysfunction

Environmental stress can interfere with the autonomic nervous system regulation of the heart, resulting in increased sympathetic nervous system excitability and/or decreased vagus nerve activity, thereby reducing heart rate variability (HRV), which is an important indicator for predicting cardiovascular events [38]. Researchers investigated the potential relationship between short-term exposure to PM (PM_1.0_, PM_2.5_, and PM_10_) and 24-hour real-time HRV in different subgroups of populations, including arrhythmia, chronic airway disease, stroke patients, residents of industrial complex areas, and the elderly. It was found that there was a significant negative correlation between single and mixed exposure to different PM indicators and HRV in all groups. Moreover, this association is more pronounced in populations with chronic airway diseases and higher exposure to air pollution. This indicates that even at relatively low environmental concentrations, PM can affect cardiac autonomic regulation through neural imbalance [39].

Sun *et al*. [40] conducted a study on a temperature control protocol for 12 male participants, which mainly involved continuous exposure to –5 °C, –10 °C, –15 °C, and –20 °C for 30 minutes. The study found that as the temperature decreased, the subjects’ Root Mean Square of Successive Differences (RMSSD), percentage of successive NN intervals differing by more than 50 ms (pNN50), and high-frequency power gradually increased, while the low-frequency to high-frequency power ratio significantly decreased, indicating enhanced vagus tone and suppressed sympathetic nervous system. Moreover, the standard deviation of the NN interval (SDNN) showed the strongest sensitivity and was linearly correlated with hemodynamic parameters (blood pressure, heart rate) and peripheral thermoregulatory markers (hand temperature), which may indicate that cold stress can temporarily improve the balance of cardiac autonomic nervous tension.

### 3.4 Prothrombotic State

Environmental stress not only induces the coagulation cascade through neural and inflammatory pathways, but also directly induces a hypercoagulable state, increasing the risk of thrombosis. During extreme heat exposure, cutaneous vasodilation and profuse sweating induce substantial fluid depletion, resulting in profound dehydration. In a retrospective cohort analysis of 43,549 Seoul residents (1995–2008), Lim *et al*. [41] identified U-curve relationships between ambient temperature and dehydration biomarkers: blood urea nitrogen-to-creatinine (BUN/Cr) ratio, urine specific gravity, plasma osmolality, and hematocrit all reached nadir values at 22–27 °C, with significant elevations beyond this thermoneutral zone. This pattern exhibited precise concordance with cardiovascular mortality trends, demonstrating that heat-induced hemoconcentration (manifested through thrombocytosis and leukocytosis) constitutes critical prothrombotic mechanisms under thermal stress. What’s more, exposure to low temperatures could cause peripheral vascular constriction, increased urination, resulting in low blood volume, blood concentration, and increased blood viscosity. This hemodynamic change could enhance hypercoagulability by synergistically increasing platelet aggregation and fibrinolysis inhibition, thereby accelerating thrombus formation in arterial and venous compartments. In comparison, a nationwide case-crossover study conducted in Sweden involving 120,380 hospitalized MI patients showed that cold wave exposure (≥2 consecutive days below the 10th percentile temperature) was associated with a 9.5% increase in STEMI onset risk (OR = 1.095; 95% CI 1.023–1.172) during lag periods of 2–6 days [19]. While both heat and cold stress can promote thrombogenesis through hemorheological and vascular changes, this finding suggests that cold exposure may confer a slightly greater short-term prothrombotic risk, particularly for acute coronary events in vulnerable individuals. Inhalation of particulate pollutants (PM_2.5_, PM_10_) and sulfur dioxide could activate alveolar macrophages and endothelial cells, release procoagulant molecules, and increase plasma coagulation factor levels [42].

### 3.5 Neuroendocrine and Psychosocial Stress Pathways

Extreme weather and climate disasters not only threaten personal safety but also have a severe impact on mental health. Previous studies have found that floods can lead to an increased incidence of post-traumatic stress disorder (PTSD) [43]. Key risk factors include severe injuries and feelings of isolation during floods, highlighting the psychosocial burden of such events and the need for targeted recovery care and long-term support. Wildfire smoke could also worsen mental health. An analysis of the 2020 California wildfire season showed that increased PM_2.5_ concentrations were associated with higher numbers of people seeking emergency care for depression, anxiety, and other mood disorders. Among these, the relative increase in visits was highest for women, children, and socially economically disadvantaged groups [44]. Additionally, hurricanes also precipitate depressive symptoms in affected communities. In Puerto Rico, high-risk adults surveyed before and after Hurricanes Maria and Irma showed that pre-disaster health vulnerabilities—including prior depression—predicted significantly elevated post-disaster major depressive disorder symptoms [45]. This emphasizes how climate shocks interact with baseline mental health status. Psychosocial stress activates the hypothalamic–pituitary–adrenal (HPA) axis and the sympathetic nervous system, leading to increased catecholamine levels, elevated heart rate, and vasoconstriction. Chronic activation can promote endothelial dysfunction, insulin resistance, and systemic inflammation—key pathways linking stress to atherosclerosis and arrhythmogenesis. Beyond mental-health sequelae, climate-induced psychological stress confers long-term cardiovascular risk. In a South African cohort of over one million adults, individuals diagnosed with PTSD had an increased risk of major adverse cardiovascular events (MACEs) during a median follow-up period of three years, indicating that trauma-related stress is an independent driver of cardiovascular disease [46]. Together, these findings emphasize that climate-induced psychological trauma may trigger cardiovascular dysfunction through prolonged neurohormonal activation, endothelial injury, and systemic inflammation.

## 4. Clinical and Epidemiological Evidence

### 4.1 Acute Cardiovascular Events

Acute environmental exposure can induce cardiovascular events within minutes to days. Multiple case cross-studies and distributed lag nonlinear model (DLNM) analyses have found a significant increase in myocardial infarction, stroke, and overall cardiovascular hospitalization rates immediately after exposure to extreme temperature and pollution peaks. The DLNM analysis conducted in the suburbs of Wuwei, China (2011–2015, 53,642 cardiovascular hospitalizations) showed that high temperatures had the strongest immediate triggering effect on daily (lag 0) cardiovascular hospitalizations, surpassing the cold effect, and women and the elderly were more sensitive [47]. Recent studies have shown that short-term exposure to PM_2.5_ can increase the risk of CVD. And through stratified analysis, it was found that females, older residents, and the acute MI group have a higher risk [48]. In addition, hourly exposure to PM_2.5_, PM_10_, NO_2_, and SO_2_ is significantly correlated with overall and ischemic stroke. This association peaks at 1 hour after exposure and lasts for about 2 h, especially in males and patients under 65 years old [49]. These findings confirm that short-term environmental exposures—especially to heat and particulate matter—can rapidly precipitate cardiovascular events. Vulnerable populations such as the elderly, women, and those with pre-existing conditions appear particularly susceptible, highlighting the need for real-time warning and protection systems.

### 4.2 Chronic CVD Progression

Long-term climate-related environmental exposure promotes the progression of chronic CVD by accelerating atherosclerosis, promoting plaque instability, and exacerbating heart failure. In a prospective cohort study involving 606 asymptomatic, low-cardiovascular risk adults, researchers evaluated the Coronary calcium score (CCS) using Agatston and spatially weighted calcification score (SWCS). The results show that long-term exposure to air pollution can trigger acute adverse events, including the impact on the development of atherosclerosis, and increase the risk of coronary artery disease (CAD) in people with low cardiovascular risk [50]. Long-term (≥2-year) residential PM_2.5_ exposure demonstrated an independent association with optical coherence tomography-verified plaque rupture (PR) (OR = 1.19, 95% CI 1.04–1.38; *p* = 0.015) [51]. Furthermore, the increase of PM_2.5_ concentration was significantly related to thin-cap fibrous atherosclerotic plaque (TCFA), macrophage infiltration (MØI), and the increase of systemic CRP indicating that chronic pollutant exposure may induce the formation and instability of atherosclerotic plaque by enhancing oxidative stress or increasing the release of proinflammatory factors. These studies collectively demonstrate that relevant environmental exposures pose delayed yet significant cardiovascular risks.

### 4.3 Regional and Temporal Trends

The impact of environmental exposure on cardiovascular health is significantly heterogeneous in geographical and temporal dimensions. Wang *et al*. [52], based on daily cardiovascular deaths and meteorological data from 136 Chinese cities between 2006 and 2019, used a time-varying distributed lag model to assess the temporal evolution of heatwaves and cold waves’ attributable risks for cardiovascular mortality. The results indicated that heatwaves can increase the mortality rate of the entire population, with the most significant increase observed in females and those aged 65–74; In contrast, the impact of cold waves seems to decrease with increasing statistical significance, which may reflect the temporal dynamics of public health responses and individual adaptability. A recent US cohort study reviewed the association between extreme heat days and adult cardiovascular mortality from 2008 to 2017. Analysis showed that extreme heat weather is significantly positively correlated with cardiovascular mortality, but the strength of this association varies in different regions; In addition, the non-elderly and elderly adults, men, women, non-Hispanic whites, and non-Hispanic blacks exhibited different sensitivities to high temperatures. With the increase of extreme heat events, this difference based on regional and population susceptibility may further intensify [53]. These regional disparities may be driven by a combination of factors. Urban areas, for example, tend to experience higher ambient temperatures due to the urban heat island effect, yet may have better access to healthcare and cooling infrastructure, which can mitigate health impacts. In contrast, rural populations may be more vulnerable due to limited medical resources and longer emergency response times [19]. Moreover, differences in housing quality, air conditioning availability, socioeconomic status, and climate adaptation policies can substantially affect how communities cope with environmental stress. For instance, low-income neighborhoods often lack green space and thermal insulation, making residents more susceptible to heat-related cardiovascular events despite similar exposure levels. Overall, the cardiovascular consequences of extreme weather events are shaped not only by environmental intensity but also by infrastructure resilience and population vulnerability. Strengthening emergency response capacity and equitable resource allocation are critical to mitigate these impacts.

### 4.4 Vulnerable Populations

The impact of environmental stress on cardiovascular health varies among different populations, and different groups face different risks due to physiological, socio-economic, and occupational factors. As people age, their ability to regulate body temperature weakens and the number of comorbidities increases, making them particularly sensitive to extreme heat. In a longitudinal cohort study of 27,233 Chinese adults aged 65 and above, it was found that the number of extremely hot days per year is associated with an increased risk of all-cause mortality, with the duration of heat waves having the most significant impact on mortality risk, at 3.80% (95% CI 2.85 to 4.74%) [54]. Moreover, heat waves were associated with increased adverse health events among dually eligible individuals 65 years and older. Long-term physical labor under high temperatures can significantly disrupt cardiovascular homeostasis. Research showed that the longer the working hours, the greater the cardiac load [55, 56]. In addition to age and occupational exposure, socioeconomic status is also a key determinant of vulnerability. Individuals residing in low-income urban communities often lack access to air conditioning, high-quality housing insulation, and adequate healthcare services, all of which exacerbate their susceptibility to heat-related cardiovascular complications. Previous studies have demonstrated that, even after adjusting for environmental exposure intensity, heat-related cardiovascular mortality remains significantly higher in economically disadvantaged counties [53]. Moreover, individuals with chronic conditions such as hypertension, diabetes, or heart failure often exhibit impaired thermoregulatory capacity and reduced physiological resilience, placing them at greater risk during heatwaves. Therefore, targeted surveillance and adaptive interventions that prioritize these high-risk groups are necessary to reduce health disparities.

## 5. Prevention and Mitigation Strategies

### 5.1 Clinical Recommendations

For patients with CAD or heart failure, the use of portable high-efficiency particulate air (HEPA) purifiers is recommended to reduce indoor PM_2.5_ exposure, particularly during pollution events. In a randomized crossover trial, continuous operation of HEPA purifiers for 48 h led to a 60–70% reduction in indoor PM_2.5_ concentrations, along with measurable improvements in vascular function and blood pressure among older adults at cardiovascular risk [57]. It is also recommended that patients with high-risk heart failure be included in a structured remote monitoring program (daily weight, blood pressure, and symptom reporting) [58]. Research has found that issuing heat wave warnings in advance reduces hospitalization rates and long-term CVD risks due to heat-related illnesses [59]. Therefore, it is recommended that the public subscribe to local heatwave alerts and take corresponding actions based on the warnings. In addition, maintaining blood volume balance is crucial for preventing hypotension and arrhythmia caused by dehydration in elderly and heart failure patients.

### 5.2 Public Health and Infrastructure Responses

Effective public health and infrastructure interventions are critical to reducing the climate-related cardiovascular burden, and several strategies have been shown to be feasible. Adams *et al*. [60] conducted spatial modeling across 81 U.S. cities and found that reallocating existing cooling centers—without increasing the total number—could improve accessibility for high-risk groups by over 25%, primarily by targeting areas with high concentrations of elderly and low-income residents. This strategy significantly reduced “cooling access deserts” and provided a quantitative framework for cost-effective urban adaptation planning [60]. At the same time, a laboratory-based thermal wave simulation study found that after entering the air-conditioned environment for 2 h at noon, the core temperature of the subjects was reduced by 0.8 °C (95% CI 0.6–0.9) compared with the control group. Although it recovered within 2 h after returning to the high-temperature environment, the experiment proved that short-term cooling could significantly relieve the acute burden of heat stress on the cardiovascular system [61]. According to a report from the Centers for Disease Control and Prevention (CDC) in the United States, the rate of heat-related emergency visits increased by 15% from May to September 2023 compared to the average level of the same period in 2018–2022, especially among males aged 18–64. This monitoring data suggests that public health institutions need to strengthen their preparation for emergency and inpatient services during heatwaves, including setting up temporary cooling and summer shelters and water replenishment stations to ensure the resilience of the healthcare system [62].

### 5.3 Policy and Co-benefits

Multiple studies have shown that policies targeting carbon emissions and urban transportation can not only mitigate climate change but also significantly improve cardiovascular health. After the implementation of Low Emission Zones/Congestion Charging Zone (LEZ/CCZ) in multiple European cities, levels of PM_10_ and NO_2_ in traffic exhaust have decreased. A meta-analysis of long-term longitudinal studies included in Lancet Public Health shows that out of five studies on LEZ, four reported a 4–7% reduction in cardiovascular hospitalization rates within 1–2 years; evaluations of the CCZ in London also found significant reductions in overall emergency admissions and cardiovascular-related injuries [63]. Further analysis of the Ultra-Low Emission Zone (ULEZ) policy in London revealed a 9.3% annual reduction in cardiovascular emergency admissions and a 5.1% decrease in all-cause emergency visits following implementation, highlighting its sustained public health impact [64]. On the other hand, forty-five cities in China have successively introduced shared bike platforms, using cross-city promotion as a quasi-experiment to analyze 8,107,363 health check-up records. The results show that six months after the introduction of shared bikes, the average systolic blood pressure decreased by 0.67 mmHg (95% CI –1.15 to –0.19), and the prevalence of hypertension reduced by 1.4 percentage points, with particularly greater benefits for younger, male, and non-obese individuals [65]. In addition to existing mitigation strategies, future policies may incorporate climate-health co-benefit tools such as carbon taxation, green urban design (e.g., reflective roofs, shade infrastructure), and smart health technologies (e.g., wearable sensors linked to remote alert systems). However, implementation must consider infrastructural disparities, privacy concerns, and the need for public education to ensure equity and effectiveness. Table 2 (Ref. [57, 58, 59, 60, 61, 63, 64, 65]) shows the benefits of environmental and policy interventions for cardiovascular health.

**Table 2.** **Cardiovascular benefits of selected environmental and policy interventions with supporting evidence**.

Intervention	Cardiovascular benefit	Reference
HEPA air purifier	Reduced indoor PM_2.5_ by ∼65%; improved BP and vascular function	Xia X *et al*. 2023 [57]
Telemonitoring for heart failure	Lowered hospitalization rate and associated costs	Kokkonen J *et al*. 2024 [58]
Heatwave warning systems	Reduced CVD risk and heat-related hospitalizations	Huang Q *et al*. 2025 [59]
Cooling center redistribution	Identified limited access for older adults; recommended optimization	Adams QH *et al*. 2023 [60]
Short-term air conditioning	Decreased core temperature by 0.8 °C; mitigated cardiovascular strain	Meade RD *et al*. 2023 [61]
Low Emission Zones (LEZs)	Reduced CVD admissions by 4–7% post-implementation	Chamberlain RC *et al*. 2023 [63]
Ultra-Low Emission Zone (ULEZ)	Decreased CVD ER visits by 9.3%; reduced all-cause ER use by 5.1%	Chamberlain RC *et al*. 2023 [64]
Shared bicycle systems	Lowered systolic BP and hypertension prevalence	Agarwal S *et al*. 2024 [65]

HEPA, High-Efficiency Particulate Air; CVD, Cardiovascular Disease; ER, Emergency Room; BP, Blood Pressure.

## 6. Research Gaps and Future Directions

Despite the significant advances in climate change and cardiovascular health in recent years, several key areas remain to be explored and innovative approaches.

### 6.1 Personal Exposure Monitoring and Spatiotemporal Resolution

Current research mostly relies on macro meteorological or air quality monitoring data, lacking personal-level exposure indicators with higher spatiotemporal resolution (such as portable sensors and satellite remote sensing combined with micro-environment models). In the future, wearable devices, mobile phone positioning, and environmental sensor networks should be integrated to construct dynamic, personalized exposure profiles, enabling more accurate analysis of the impact of short-term and long-term exposures on cardiovascular function. For example, the MicroPEM™ device has been validated under high-pollution conditions in Beijing, showing good agreement with reference-grade samplers across a PM_2.5_ range of 6 to 461 µg/m^3^, confirming its suitability for personal exposure monitoring in real-world urban environments [66].

### 6.2 Crossing Action and Comprehensive Model of Multiple Pathologies

Heat stress, pollution, and psychosocial stress often coexist, and most of the existing studies focus on a single stressor. It is necessary to develop a comprehensive risk assessment model for multi-factor combined exposure to reveal the interaction and superposition effect between different pathways (neuro-endocrine, inflammation-oxidation, coagulation). Applied systems biology and machine learning techniques to integrate omics (genetic, epigenetic, metabolite) and physiological signal data to construct an interpretable “exposure pathway outcome” multi-scale network.

### 6.3 Mechanisms for Vulnerable Populations and Health Inequalities

Vulnerable groups such as minorities, low-income, and patients with chronic diseases are more severely affected, but there is still limited empirical data on gene–environment interactions, thermoregulatory variability, and stress response differences across vulnerable subpopulations. Future research should focus on the interaction between social determinants and environment, carry out community-based participatory research, and develop intervention strategies that are culturally and resource-appropriate.

### 6.4 Future Risk Projections Under Climate Change Scenarios

Most of the current retrospective analyses are based on past climate and health data, and there is a lack of studies that incorporate future extreme event frequency and intensity from global climate model (GCM) outputs into health impact projections. Interdisciplinary collaboration should be promoted to integrate climate science, epidemiology, and public health models to achieve forward-looking predictions of the burden of CVD under different carbon emission and adaptation pathways.

### 6.5 Long-term Cohort and Longitudinal Mining of Large-scale Electronic Health Records

As biobanks and electronic health records (EHR) continue to grow, larger prospective cohort studies can be conducted to systematically explore the association between exposure dynamics and disease progression. International cooperation and data sharing should be strengthened to build a climate-health database and promote open science and reproducible research. However, persistent ethical and technical challenges must be addressed, particularly regarding data privacy protection, standardization of EHRs, and representative sampling of EHR-derived populations. The development of comprehensive governance frameworks is imperative to ensure both data security and equitable utilization in climate-health research.

## 7. Conclusion

This review comprehensively examines the multiple pathways through which climate change affects cardiovascular health: starting from environmental stressors such as heatwaves, extreme weather events, air pollution, and psychological stress, these factors trigger various biological mechanisms, including thermoregulation and hemodynamic stress, inflammation and oxidative stress, autonomic nervous system imbalance, and pro-coagulant state. Ultimately, these mechanisms lead to acute events like acute MI and stroke, as well as accelerated clinical outcomes of chronic conditions such as atherosclerosis and heart failure. Large-scale epidemiological evidence shows that risks not only vary rapidly with exposure intensity and duration but also exhibit significant differences across regions, times, and populations (such as the elderly, patients with chronic diseases, and those with low socioeconomic status).

In terms of response strategies, clinically, there should be active promotion of indoor air purification, remote monitoring, early warning systems, and individualized fluid and medication adjustments; at the public health and infrastructure level, heat alerts, cooling center configurations, and urban greening need to be optimized; policy formulation can achieve synergistic benefits for carbon reduction and cardiovascular health by phasing out high-pollution energy sources, restricting traffic in congested areas, and encouraging active travel. Among these interventions, large-scale policy initiatives—such as low-emission zones and active transport infrastructure—demonstrate the greatest population-level health impact, supported by sustained reductions in cardiovascular hospitalizations and mortality. Clinical interventions (e.g., HEPA filtration, telemonitoring) provide targeted protection for vulnerable individuals, while public health measures (e.g., cooling centers, alerts) enhance resilience during acute events. Such prioritization helps guide resource allocation and policy action. Future research should focus on high-resolution exposure assessment, integration of multi-factor pathways, empirical validation of mechanisms of health inequality and intervention effects, and long-term risk prediction in conjunction with climate models.

In general, to address the cardiovascular health challenges posed by climate change, a combination of interdisciplinary collaboration, evidence-based practice, and systematic interventions is needed to reduce the burden of CVD, protect vulnerable populations, and provide solid support for global health and sustainable development.
